# Structure and Pathology of Tau Protein in Alzheimer Disease

**DOI:** 10.1155/2012/731526

**Published:** 2012-05-29

**Authors:** Michala Kolarova, Francisco García-Sierra, Ales Bartos, Jan Ricny, Daniela Ripova

**Affiliations:** ^1^Laboratory of Biochemistry and Brain Pathophysiology and AD Center, Prague Psychiatric Center, Ústavní 91, 181 03 Prague 8, Czech Republic; ^2^Third Faculty of Medicine, Charles University in Prague, Ruská 87, 100 00 Prague 10, Czech Republic; ^3^Department of Cell Biology, Center of Research and Advanced Studies, National Polytechnic Institute, Avenue Instituto Politecnico Nacional 2508, 07360 Mexico City, DF, Mexico; ^4^Department of Neurology, Third Faculty of Medicine, Faculty Hospital Královské Vinohrady, Charles University in Prague, Šrobárova 50, 100 34 Prague 10, Czech Republic

## Abstract

Alzheimer's disease (AD) is the most common type of dementia. In connection with the global trend of prolonging human life and the increasing number of elderly in the population, the AD becomes one of the most serious health and socioeconomic problems of the present. Tau protein promotes assembly and stabilizes microtubules, which contributes to the proper function of neuron. Alterations in the amount or the structure of tau protein can affect its role as a stabilizer of microtubules as well as some of the processes in which it is implicated. The molecular mechanisms governing tau aggregation are mainly represented by several posttranslational modifications that alter its structure and conformational state. Hence, abnormal phosphorylation and truncation of tau protein have gained attention as key mechanisms that become tau protein in a pathological entity. Evidences about the clinicopathological significance of phosphorylated and truncated tau have been documented during the progression of AD as well as their capacity to exert cytotoxicity when expressed in cell and animal models. This paper describes the normal structure and function of tau protein and its major alterations during its pathological aggregation in AD.

## 1. Introduction

Alzheimer's disease (AD) is the most common type of dementia characterized by memory impairment and alteration of diverse cognitive abilities. In association with the global trend of prolonging human life and increasing number of elderly in the human population, AD becomes one of the most important health and socioeconomic problems of the present. AD and related tauopathies are histopathologically characterized by slow and progressive neurodegeneration, which is associated mostly with intracellular accumulation of tau protein leading to the so-called neurofibrillary tangles (NFTs) and other inclusions containing modified tau [1]. Tau protein was discovered in the mid-1970s of the 20th century by studying factors necessary for microtubule formation. Tau protein promotes tubulin assembly into microtubules, one of the major components of the neuronal cytoskeleton that defines the normal morphology and provides structural support to the neurons [2]. Tubulin binding of tau is regulated by its phosphorylation state, which is regulated normally by coordinated action of kinases and phosphatases on tau molecule [3, 4]. In pathological conditions, such as the case in AD, not only does abnormal phosphorylation of tau protein decrease its tubulin binding capacity leading to microtubule disorganization, but also this protein self-polymerizes and aggregates in the form of NFTs [5, 6].

## 2. The Tau Gene

The human tau gene is located over 100 kb on the long arm of chromosome 17 at band position 17q21 and contains 16 exons. Exon 1 is part of the promoter and is transcribed but not translated. Exons 1, 4, 5, 7, 9, 11, 12, and 13 are constitutive exons. Exons 2, 3, and 10 are alternatively spliced and manifesting in the adult brain. Exon 2 can appear alone, but exon 3 never appears independently of exon 2 [7]. In the central nervous system, alternative splicing of exons 2, 3, and 10 results in the appearance of six tau isoforms that are differentially expressed during development of the brain [7].

## 3. Structure and Function of Tau Protein

**Figure 1:**
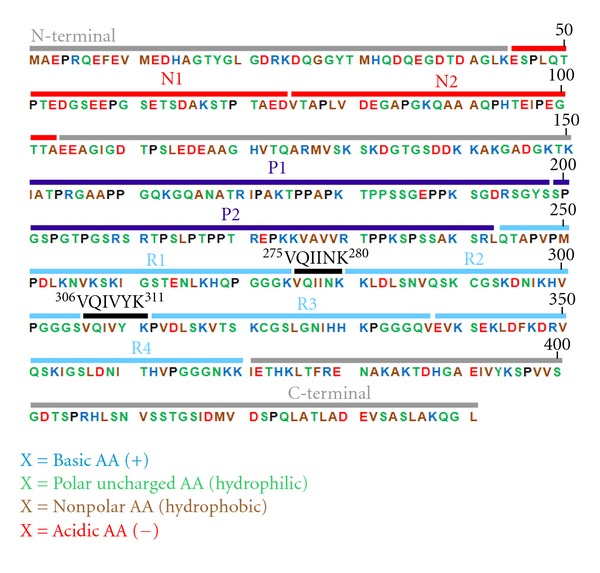
Amino acid sequence of the longest tau isoform (441 amino acids). N1 and N2: the polypeptide sequences encoded by exons 2 and 3; P1 and P2: proline-rich regions; R1–R4: microtubule-binding domains encoded by exons 9–12; ^275^VQIINK^280^ and ^306^VQIVYK^311^: sequences with *β*-structure (modified by [13]).

Tau protein belongs to a group of proteins referred to as Microtubule-Associated Proteins (MAPs), that in common are heat resistant and limited affected by acid treatment without loss their function [8]. This property observed in tau is due to a very low content of secondary structure. In fact, a number of biophysical studies revealed that tau is a prototypical “natively unfolded” protein [9–11]. Since disordered proteins tend to be highly flexible and have variable conformations, they have not been amenable for structure analysis by crystallography so far. Thus nuclear magnetic resonance spectroscopy is the only plausible method that allows a description of their conformations and dynamics with high resolution [12]. Now it is possible to obtain the complete backbone assignment of 441-residue tau (the longest tau isoform found in the human central nervous system; Figure 1). This makes it possible to probe the structure and dynamics of the full-length soluble protein and determine the residues involved in the interaction between tau and microtubules at single residue resolution [13].

Six isoforms of tau protein differ according to the contents of three (3R) or four (4R) tubulin binding domains (repeats, R) of 31 or 32 amino acids in the C-terminal part of tau protein and one (1N), two (2N), or no inserts of 29 amino acids each in the N-terminal portion of the molecule. These isoforms, which vary in size from 352 to 441 amino acid residues, are related to the presence or absence of sequences encoded by exons 2, 3, or 10. Inclusion of the imperfect repeat region encoding exon 10 leads to the expression of tau containing four microtubule-binding repeats (MTBRs) (4R tau: 0N4R, 1N4R, 2N4R), while exclusion of exon 10 results in splicing products expressing tau with three MTBRs (3R tau: 0N3R, 1N3R, 2N3R) [7, 14]. These six isoforms are also referred to as *τ*3L, *τ*3S, *τ*3, *τ*4L, *τ*4S, and *τ*4 [15]. Primary sequence analysis demonstrates that tau consists of a half-N-terminal acidic portion followed by a proline-rich region and the C-terminal tail, which is the basic part of the protein. The polypeptide sequences encoded by exons 2 and 3 add acidity to tau, whereas exon 10 encodes a positively charged sequence that contributes to the basic character of tau protein. On the other hand, the N-terminal region has an isoelectric point (pI) of 3.8 followed by the proline-rich domain, which has a pI of 11.4. The C-terminal region is also positively charged with a pI of 10.8. In other words, tau protein is rather a dipole with two domains of opposite charge, which can be modulated by posttranslational modifications [16]. Because each of these isoforms has specific physiological roles, they are differentially expressed during the development of the brain. For instance, only one tau isoform, characterized by 3R and no N-terminal inserts, is present during fetal stages, while the isoforms with one or two N-terminal inserts and 3- or 4R are expressed during adulthood [7].

Tau protein is present in a greater extent in axons from neurons, but it also occurs in the oligodendrocytes. Another microtubule-binding protein referred to as MAP2 is located in the somatodendritic compartment of neurons, whereas MAP4 is much ubiquitous [17].

### 3.1. The Projection Domain and Its Interaction with Other Molecules

The two 29-amino-acid sequences encoded by exons 2 and 3 give different lengths to the N-terminal part of tau protein. The N-terminal part is referred to as the projection domain since it projects from the microtubule surface where it may interact with other cytoskeletal elements and the neuronal plasma membrane. In fact, the projection domains of tau protein determine spacing between microtubules in the axon and may increase the axonal diameter [7, 18]. Peripheral neurons often project a very long axon with a large diameter. This type of neurons contains an additional N-terminal tau sequence encoded by exon 4A and so generates a specific tau isoform called “big tau” [7, 18–20]. As to the interactions with other cytoskeletal components, tau protein binds to spectrin and actin filaments, which may allow tau-stabilized microtubules to interconnect with neurofilaments that restrict the flexibility of the microtubule lattices. Another molecule that interacts with tau protein is a peptidyl-prolyl cis/trans isomerase Pin 1. It isomerizes only phosphoserine/threonine-proline motifs and binds to the tau protein after its phosphorylation on Thr^231^ residue. Isomerization induces conformational changes that make tau accessible for Protein Phosphatase (PP) 2A, which in turn leads to tau dephosphorylation. Protein Pin 1 regulates functions of tau protein and APP and is important for protection against the degeneration that occurs during the ageing process. Activity of Pin 1 is decreased by oxidation in AD [21]. Moreover, tau protein through its N-terminal projection domain may interact with intracellular membranous elements such as the mitochondria [22] and the neuronal plasma membrane [23]. In the cytosol of neurons the pools of tau protein in either phosphorylated or dephosphorylated forms are maintained in equilibrium by coordinated actions of kinases and phosphatases, respectively. Several studies in cell lines revealed that tau protein bound to the plasma membrane is dephosphorylated [24, 25]. Tau protein binds through its proline-rich region to the Src-homology 3 (SH3) domains of several proteins, including Fyn, a tyrosine kinase from the Src-family. The association of tau and Fyn depends on the phosphorylation state of tau, because insoluble PHF-tau isolated from AD brain does not bind to the Fyn SH3 domain [26]. Recently, Fyn has been demonstrated to play a role in protein trafficking [27]. For example, Fyn can increase the surface expression of the amyloid precursor protein (APP) through tyrosine phosphorylation [28]. The trafficking of tau protein to the plasma membrane is a bidirectional process, because increased tau phosphorylation induced by PP2A inhibition significantly reduces the proportion of membrane-associated tau. The active relocalization of tau in response to changes in phosphorylation suggests a possible role of this protein in intracellular signaling pathways [29, 30]. It was recently shown that tau binds to the Fyn in dendritic spines, and this interaction regulates N-methyl-D-aspartic acid (NMDA) receptor signaling [31]. Pathological tau may participate in the localization of Fyn kinase to the postsynaptic compartment, where it phosphorylates NMDAR subunits, causing increased inward Ca^2+^ conductance and leading to excitotoxicity [32]. *In vivo*, tau has been demonstrated to interact directly with ionotropic glutamate receptors [33]. In oligodendrocytes, the association of tau with Fyn regulates the outgrowth of cytoplasmic process [34]. Impaired interaction of Fyn kinase and hyperphosphorylated tau protein leads to hypomyelination and evolving demyelination of axons [34]. All these evidences indicate that the phosphorylated state of tau protein not only affects microtubule stability but also produces alterations on neuronal plasticity.

### 3.2. The Domain Associated with the Microtubules

**Figure 2:**
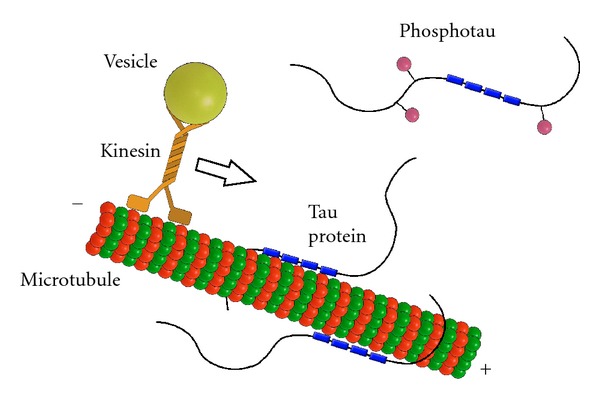
Normal function of tau protein. Tau protein stabilizes microtubules through four tubulin binding domains (blue boxes) in case of the longest isoform. Binding of tau protein to the microtubules is maintained in equilibrium by coordinated actions of kinases and phosphatases. The phosphorylation of tau (pink balls) regulates its activity to bind to microtubules and can affect axonal transport. Tau protein may inhibit the plus-end-directed transport of vesicles along microtubules by kinesin.

Tau protein binds microtubules through some repeated domains (R1–R4) (encoded by exons 9–12) located at the C-terminus of the molecule (Figure 2) [35]. Each repeat consists of stretches of a highly conserved 18 residues that are imperfectly repeated three times in the fetal tau protein and four times in the adult specific form [35]. The repeats are separated from each other by 13- or 14-residue spacer regions. The main function of tau, aforementioned as a promoter of tubulin polymerization, depends mostly on the MTBR [35, 36]. It has been reported that *in vitro* tau protein increases the rate of microtubule polymerization and concomitantly inhibits its rate of depolymerization [37]. The 18-amino-acid repeats bind to microtubules through a flexible array of distributed weak sites. The adult form of tau promotes assembly of microtubules more actively than fetal forms [14, 38]. Interestingly, the most potent part that induces microtubule polymerization is the interregion between repeats 1 and 2 (R1-R2 interregion) and more specifically the peptide ^275^KVQIINKK^280^ within this sequence [7, 39]. This R1-R2 interregion is unique to 4R tau, adult specific, and responsible for the difference in the binding affinities between 3R and 4R tau [7, 35]. Recent evidence supports a role for the MTBR in the modulation of the phosphorylation state of tau protein. A direct and competitive binding has been demonstrated between this region (residues 224–236 according to the numbering of the longest isoform) and the microtubule on one hand and the same region with the PP2A on the other hand [40]. As a consequence, microtubules could inhibit PP2A activity by competing for binding to tau at the MTBR.

Microtubules contribute to diverse cellular processes such as cell morphogenesis, cell division, and intracellular trafficking [41, 42]. In cells, microtubules can change their lengths via dynamic instability [43]. They can serve as tracks for organelle transport mediated by microtubule-dependent motor proteins such as the plus-end-directed motor kinesin and its relatives, or the minus-end-directed motor dynein [44, 45]. These motors can transport their cargoes, for example, mitochondria [46, 47], lysosomes [48], peroxisomes [49], and endocytotic or exocytotic vesicles [50] towards the cell periphery or back towards the microtubule organizing center (MTOC), respectively. It has been shown that tau protein affects axonal transport [17, 51, 52]. Tau protein alters intracellular traffic due to its tight binding to microtubules and probably detaches the cargoes from kinesin. Nevertheless, tau protein has no influence on speed of kinesin with cargoes [52]. This implies that the phosphorylation of tau should play an important role because this modification regulates tau's affinity to microtubules.

## 4. Tau Pathology

In AD, the normal role of tau protein is ineffective to keep the cytoskeleton well organized in the axonal process because this protein loses its capacity to bind to microtubules. This abnormal behavior is promoted by conformational changes and misfoldings in the normal structure of tau [53–55] that leads to its aberrant aggregation into fibrillary structures inside the neurons of demented individuals [56–58]. Thus, most of the altered pools of tau protein in the disease are redistributed and aggregated in both the somatodendritic compartment and isolated processes of affected neurons. Alterations in the amount or the structure of tau protein can affect stabilization of microtubules and other processes related to this protein [59, 60].

For instance, overexpression or mislocalization that increase intracellular concentration of tau may inhibit the plus-end-directed transport of vesicles along microtubules by kinesin so that the minus-end-directed transport by dynein becomes more dominant [17]. Inhibition of transport to the plus-end of microtubule slows down exocytosis and affects the distribution of mitochondria which become clustered near to the MTOC. The absence of mitochondria and endoplasmic reticulum in the peripheral regions of the axons could produce a decrease in glucose and lipid metabolism and ATP synthesis and loss of Ca^2+^ homeostasis [61] that leads to a distal degeneration process referred to as “dying back” of axons [62]. Moreover, phosphorylated tau protein has affinity to the kinesin and therefore is transported to the distal sites of neuropil. This may account for the observation that tangle pathology in AD appears to initiate distally and then spreads in a retrograde fashion to the perikaryon. This process may be a mechanism to protect the stability of the microtubules by transporting hyperphosphorylated tau more rapidly to other cellular locations where tau can form aggregates [51].

The mechanisms by which tau protein becomes a nonfunctional entity are in debate. Abnormal posttranslational modifications are proposed to be the main cause of this failure [63, 64]. In this regard, abnormal phosphorylation (hyperphosphorylation), acetylation, glycation, ubiquitination, nitration, proteolytic cleavage (truncation), conformational changes, and some other modifications [53, 65–73] have been proposed to cause the loss of normal function and the gain of pathological features of tau protein. In the upcoming sections we will focus our interest to describe evidence supporting abnormal phosphorylation, acetylation, and truncation of tau as major changes during the pathological processing of tau protein in AD.

### 4.1. The Hyperphosphorylation of Tau Protein

The phosphorylation of tau regulates its activity to bind to microtubules and stimulate their assembly as previously outlined. A normal level of phosphorylation is required for the optimal function of tau, whereas the hyperphosphorylated state makes tau to lose its biological activity. Regarding the potential propensity of tau protein to be phosphorylated, it was reported that the longest variant of tau protein (441 amino acid) holds about 80 potential serine or threonine phosphorylation sites [7]. Most of these potential sites are located at the vicinity of the MTBR in the proline-rich region and in the C-terminal extreme of the molecule of tau protein [16, 74] with the exception of Ser^262^, Ser^293^, Ser^324^, and Ser^356^ (motif KXGS) in R1, R2, R3, and R4 domains [75, 76]. In the disease the abnormal phosphorylation of tau could be, but not mutually exclusive, the result of upregulation of tau kinase(s) or downregulation of tau phosphatase(s) [62, 74]. A number of these enzymes have been evaluated and those kinases that are believed to play the most important role in phosphorylation of tau in the brain include GSK-3*β*, cyclin-dependent kinase 5 (cdk5), cAMP-dependent protein kinase (PKA), and calcium/calmodulin-dependent kinase II (CaMK-II) [77]. GSK-3*β* may play major role in regulating tau phosphorylation in both physiological and pathological conditions. GSK-3*β* can phosphorylate tau on Ser^199^, Thr^231^, Ser^396^, Ser^400^, Ser^404^, and Ser^413^ *in vivo* and *in vitro* (numbered according to the longest tau isoform), residues that are mostly phosphorylated in PHF-tau [78]. Aforementioned phosphorylation at Thr^231^ causes a local conformational change that allows the access of GSK-3*β* or other kinases to further phosphorylate tau. On the other hand, a complementary and opposite effect is for PP1, PP2A, PP2B, and PP2C that can dephosphorylate tau protein *in vitro *[79]. The activity of PP2A has been found to be reduced in selected areas of the brain of AD patients [4]. Overall tau phosphoprotein is at least three- to fourfold more hyperphosphorylated in the brain of AD patients than that in the brain of aged nondemented individuals [80].

At cellular level, abnormal phosphorylation of tau introduces alterations in several processes which are directly regulated by the suitable organization of the microtubule network. In a normal mature neuron, tubulin is present in over tenfold excess of tau, and thus practically all tau protein is microtubule bounded in the cell [81, 82]. In neurons affected in AD, abnormally phosphorylated cytosolic tau (AD P-tau) neither binds to tubulin nor promotes microtubule assembly [83–85]. Instead, this protein inhibits the assembly and disrupts the microtubule organization [83]. Moreover, it was reported that abnormally phosphorylated tau protein disengages normal tau from microtubules into the cytosolic phase [83], as much as 40% of the abnormally hyperphosphorylated tau in the brain of AD patients is present in the cytosol and not polymerized into paired helical filaments (PHFs) or forming NFTs [80]. The AD P-tau also removes the other two major neuronal MAPs, MAP1 and MAP2, from microtubule lattice [86]. This toxic feature of the AD P-tau appears to be solely due to its abnormal phosphorylation state because dephosphorylation of AD P-tau rescues this protein to perform its normal tasks [84].

By using a phosphorylation-dependent monoclonal antibodies against tau and mass spectrometry, it was reported that at least 39 phosphorylated sites in the tau molecule are associated with native PHF isolated from the brain of AD patients [87].

As to the *in situ* aggregation of hyperphosphorylated tau, a bunch of evidence has been generated over the years to identify abnormally phosphorylated tau as the major component of distinct neuropathological hallmarks that defines AD [6, 15, 65, 88–90]. Hyperphosphorylated tau has been observed as the major component of PHFs and straight filaments (SFs), NFTs, neuropil threads (NTs), and plaque-associated dystrophic neurites in the brain of AD cases [81, 91]. The density of NFTs distributed along the hippocampus, entorhinal cortex, and neocortex has been correlated with the degree of dementia in this disorder [92]. Moreover, the earliest accumulation of tau in the hippocampus of AD patients, prior to the formation of NFTs, has been viewed as a diffuse granular material immunoreactive to phosphorylation-dependent tau antibodies [93–95]. However, in the abnormal formation of PHFs, tau molecules may follow different alterations from which abnormal phosphorylation (although this may not be essential) causes misfolding and conformational changes that strength its abnormal aggregation [79, 96].

Recent studies demonstrated that hyperphosphorylation of tau occurs before its cleavage [97, 98] and that tau cleavage takes place before NFT formation [99]. In an *in vitro *model of ethanol-induced neuronal apoptosis, hyperphosphorylation of tau occurs before tau cleavage [98, 100]. Altogether, these results may indicate that abnormal phosphorylation is a key event that triggers the pathological aggregation of tau in AD.

### 4.2. The Acetylation of Tau Protein

The mechanism leading normal soluble tau to become hyperphosphorylated and disengaged from microtubules to form tau inclusions remains unknown and posttranslational modifications other than phosphorylation could regulate tau function and aggregation. Notably, reversible lysine acetylation has emerged as a potential regulatory modification implicated in AD and other neurodegenerative disorders. Recent studies demonstrate tau acetylation as a posttranslational modification that may regulate normal tau function [73, 101, 102]. Since acetylation neutralizes charges in the microtubule-binding domain, aberrant acetylation might interfere with the binding of tau to microtubule, leading to tau dysfunction, and suggests a role in pathological tau aggregation in AD and related tauopathies [73]. Increased tau acetylation on Lys^280^ could impair tau interactions with microtubules and provide increased pools of cytosolic tau available for pathological PHF aggregation [39, 101]. Consistent with this, Lys^280^, located in the interrepeat region (^275^VQIINKK^280^), was identified previously as one of three lysine residues most critical in modulating tau-microtubule interactions [39]. Acetylation of tau aggregates was associated with hyperphosphorylated, ThS-positive tau inclusions in both Tg mouse models and human tauopathies [101]. This implies that negative regulation of tau function could occur via phosphorylation and acetylation events alone or in combination. The molecule of tau protein contains a lot of phosphorylation sites, as mentioned previously, and most of them occur in regions flanking the microtubule-binding repeat [74], in which Lys^280^ is located. Thus, tau hyperphosphorylation may render this residue available for subsequent acetylation, which would further impair microtubule binding and/or promote tau aggregation as well as further drive pathological alterations of tau. Although protein acetylation has been extensively studied in the context of histones and gene transcription, proteomics approaches have identified acetylated proteins in the cytoplasm and other organelles [103]. Recent study suggests that acetylation of Lys^280^ may be an intermediate step in tangle formation [102]. Acetylated Lys^280^ was mostly associated with intracellular neurofibrillary tangles compared to pretangles or extracellular ghost tangles throughout all Braak stages [73, 102]. Acetylated Lys^280^ also colocalizes with N- and C-terminal specific antitau epitopes. This indicates that it is present in neurofibrillary tangles prior to subsequent tau truncation [102].

Enzymes that add an acetyl group to the protein are called histone acetyltransferase (HAT) or lysine acetyltransferase. Of four major classes of HATs, p300/CBP (protein of 300 kDa and CREB-binding protein) and pCAF (p300-associated and CBP-associated factor) are exclusively present in metazoans [104]. Enzymes that remove an acetyl group from the protein are called histone deacetylase (HDAC) or lysine deacetylase. There are three classes of HDACs. The activities of HDACs in classes I and II (HADC1–11) depend on zinc as a cofactor; the activities of class III HDACs (sirtuins) depend on the relative levels of NAD+ and NADH [105, 106]. Of the seven members of mammalian sirtuins (SIRT1–7), SIRT1 is the most studied and is strongly implicated in aging-related diseases, including AD [107]. SIRT1 levels are reduced in AD brains, and the reduction correlates with the accumulation of hyperphosphorylated tau aggregates [108]. SIRT1 was found to reduce A*β* generation by activating transcription of a gene encoding *α*-secretase [109]. SIRT1 deficiency could also exacerbate the accumulation of A*β*, which could increase tau acetylation and tau phosphorylation even further. Since a decrease in SIRT1 activity can clearly have deleterious effects on neuron health, therapeutic strategies aiming at increasing sirtuins activity in AD brain warrant further research.

### 4.3. The Aggregation of Tau Protein In Vitro

**Figure 3:**
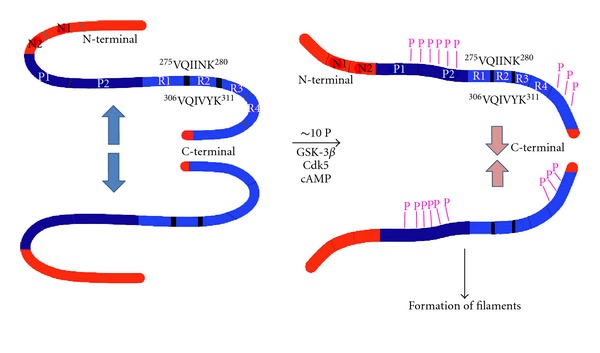
Phosphorylation of tau protein. Tau self-assembles mainly through the microtubule binding domains/repeat R3 in 3R tau proteins and through R3 and R2 in 4R tau proteins (R2 (^275^VQIINK^280^) and R3 (^306^VQIVYK^311^) have *β*-structure). N-terminal and C-terminal regions to the repeats are inhibitory. Hyperphosphorylation of tau neutralizes these basic inhibitory domains, enabling tau-tau interaction (phosphorylation sites indicated by violet Ps) (modified by [15]).

The molecule of tau has long stretches of positively and negatively charged regions that are not conducive for intermolecular hydrophobic association [81, 110]. The *β*-structure in monomeric tau is concentrated only in R2 (exon 10) and R3 (exon 11), which can self-assemble by their own into filaments [111] and coassemble with heparin as an artificial inducer [112]. Evidence *in vitro *has revealed that self-aggregation of tau into filaments is inhibited by the presence of intact N- and C-termini, which lie down over the MTBR and avoid the interaction between these sticky domains [15]. Abnormal phosphorylation of the N-terminal and the C-terminal flanking regions may induce a relaxed structural conformation in the tau molecule that unclip both extremes from the MTBR region. This situation allows the self-interaction between these sticky domains in the formation of PHF/SF (Figure 3) [15].

 Some other modifications such as deamidation could facilitate polymerization of tau protein. Curiously, several years later, it was shown that deamidation occurs in tau obtained from PHF [113]. Because a high concentration of tau protein is needed to polymerize [114], some suggest that other compounds, acting as cofactors, could be necessary to facilitate the self-assembly of tau protein [115–117]. Regardless of the phosphorylation state of tau protein, it was found that sulfoglycosaminoglycans (sGAGs), a class of polyanionic molecules, facilitate the polymerization of tau *in vitro *[115, 116]. Moreover, these sGAGs were found along with tau in NFTs, when the tau-neurofibrillary pathology was analyzed in the brain of AD cases [115, 116]. *In vitro*, tau polymerization paradigms also have utilized arachidonic acid as a polyanionic inducer [118], resulting in increased rates of filament formation. Other native polyanions such as the glutamic acid-rich region present at the C-terminal region of tubulin can also facilitate the aggregation of tau protein. This aggregation requires the presence of the third tubulin binding motif of the tau molecule [115]. Oxidation is another process that facilitates the aggregation of tau protein. Because 3R tau molecules contain only one cystein, oxidation of cystein produces disulfide cross-linking and thus self-assembly of tau protein [119]. It does not occur in 4R tau molecules with two cysteins, which may form intramolecular disulfide bonds [119].

Despite *in vitro* formed tau polymers have been demonstrated by spectroscopy, laser scattering, and electron microscopy [120–123], recent findings demonstrate that prefibrillar tau oligomers can be formed *in vitro* by light-induced cross-linking of tau with benzophenone-4-maleimide (B4M) [123]. These oligomers of tau were also observed *in situ* at the early stages of AD, when a monoclonal and specific antibody to these oligomeric entities of tau was assessed in the brain of AD cases [123]. Oligomeric species of tau protein are reported to have increased toxicity over soluble and high-ordered fibrillary aggregates such as NFTs [124–126]. In transgenic mice that overexpress tau, most of the observed cognitive alterations emerged at stages of profound occurrence of multimeric aggregates of tau and prior to the formation of NFTs [126].

### 4.4. The Truncation of Tau Protein

Proteolytic cleavage of tau protein, as an alternative mechanism involving in the abnormal aggregation of tau, was early proposed by Whischik's group at Cambridge University after extensive biochemical analysis of the minimal structure of the PHFs [69, 127, 128]. The minimal component of PHFs, referred to as the PHF core, was mostly composed of a fragment of tau only containing the region of the MTBR and ending at the position Glu^391^. Until today, identification of the enzyme that produces this proteolytic cleavage is uncertain. However, the presence of this truncation associated with the neurofibrillary pathology has been demonstrated in the brain of AD patients [129, 130]. Furthermore, from *in vitro* paradigms of polymerization, tau constructs lacking the carboxy tail assembled much faster and to a greater extent than full length tau [131]. Despite these early evidences, attention was not focused for a while on the proteolysis of tau, and its contribution to the disease was uncertain. New findings show aberrant proteolysis in the brain of AD cases associated with programmed cell death [132, 133]. Further studies were dedicated to investigate the contribution of apoptosis and associated caspases into the neurodegenerative process underlying AD. In this regard, apoptotic cells were observed to proliferate in areas of the brain that were affected by fibrillary accumulation of tau protein and amyloid-*β* deposits [134–136]. Concomitantly, increased expression of several enzymes of the family of caspases was reported in the brain of AD cases [99, 137, 138].

Caspases are cysteine proteases that cleave aspartic acid residue in the canonical consensus sequence DXXD on the carboxy side of molecule. These enzymes participate in a proteolytic cascade leading to cell death via apoptosis. The major killer caspase in neurons is caspase 3 [139]. Members of the caspase family play a critical role in A*β*-induced neuronal apoptosis [140] and are activated in apoptotic neurons in AD [141]. It was known that tau protein contains several canonical sites for caspase cleavage [142, 143], from which a susceptible residue at Asp^421^ was reported to be cleaved *in vitro* by caspase 3 [72]. The cleavage at Asp^421^ released a discrete peptide (Ser^422^-Leu^441^) that is capable of forming an amphipathic *α*-helix [144]. Tau protein truncated at Asp^421^ assembled more readily than the full-length molecule [72, 144]. When a synthetic peptide comprising the fragment after caspase cleavage was added back to the tau molecule in a polymerization paradigm, assembly of this protein was inhibited.

In the disease, the occurrence of truncation of tau protein at Asp^421^ was corroborated in association with the neurofibrillary pathology by using the monoclonal antibody Tau-C3, which specifically recognizes this cleavage site generated by caspase 3 activity [72, 145]. Interestingly, phosphorylation of tau protein at residue Ser^422^ seemed to prevent the proteolytic cleavage of tau at Asp^421^ [146]. After truncation at Asp^421^ another cleavage of tau protein has been reported to occur at Glu^391^. This state is recognized by antibody MN423, which indicates the transitions to “late” tangles [56, 67, 145]. Another truncation in the N-terminus of tau protein has been reported to occur at the residue Asp^13^, which in this case is produced by caspase 6 activity [147]. Despite the *in vitro* demonstration that this truncation at the N-terminus is important to favor tau aggregation, its pathological meaning and occurrence in the brain of AD patients is still far from proven.

The pathological effect of C-terminus truncated tau over the normal functioning of the cells has been assessed in cultured cells and transgenic animal models. By using neuronal and nonneuronal cells, overexpression of truncated tau protein produces several alterations in the organization and functioning of membranous organelles, such as mitochondria and the endoplasmic reticulum. Even some examples of cell death by apoptotic mechanisms also have been reported [148–156]. In transgenics animals, truncated-tau carrying rodents have developed alterations in cognitive performance associated with neuronal death and abnormal aggregation of cleaved tau [100, 157–162].

Finally, the abnormal role of truncation of tau protein and its pathological significance in AD has been demonstrated by clinicopathological studies where the occurrence of truncated tau associated with fibrillary structures was analyzed during the development of the dementia [130, 145, 163]. These studies corroborate the importance of the truncated tau protein at both sites Asp^421^ and Glu^391^. A positive correlation of these events with neuropathological progression of the disease was described by H. Braak and E. Braak [164] and a relationship to the clinical severity of dementia was demonstrated [130, 163]. Moreover, the presence of the Apolipoprotein-E (*ε*4) allelic variant was found in cases with an increased density of NFTs composed by the two variants of truncated tau [163].

In the hippocampus of AD patients, the maturation of NFTs is reported to be unsynchronized. Therefore these structures have different stages of tau processing [163]. It was reported that different populations of NFTs in the same hippocampal area were mutually exclusive when they were composed of either Asp^421^- or Glu^391^-truncated tau with no colocalization at any single point during the maturation of the NFTs [163]. During the progression of the disease, Asp^421^-truncation is an early event that precedes the second truncation of the C-terminus at the Glu^391^, the later occurring from intermediate to advanced stages of NFTs evolution [163]. A recent report indicates that tau protein in NFTs may be dually subjected to both apoptotic and proteosomal proteolysis since strong ubiquitination was found in Asp^421^-truncated tau associated with the neurofibrillary pathology in AD [165].

By combining of antibodies that map different regions of the molecule of tau, a continuous and specific pathway of conformational changes and truncation of tau protein has been proposed to occur during the maturation of NFTs. These antibodies are, namely, conformational and phosphorylation-dependent and recognizing truncation sites [66, 67, 145].

These studies proposed that not only the number of NFTs but also the state of proteolysis of the C-terminus which is associated with conformational changes (structural modification along the tau molecule) defines the progression of AD [166]. All these findings together may support the relevance of truncation of tau protein as a pathogenic event and reliable marker for both diagnosis and therapeutic targeting in AD.

## 5. Conclusion

It is largely accepted that clinical manifestation of dementia in AD is due to the neuronal loss occurring in those areas of the brain associated with cognitive functions of the patients. Fibrillary inclusions are reported to be responsible for cell death. However, discrepancy has emerged from studies demonstrating that cognitive impairment in animal models occurs earlier than the initial formation of fibrillary structures. Extrapolation of these results to the real onset of the disease in humans is still considered inaccurate for some researchers. In this regard, a bunch of reports analyzing the brain of AD patients come to an agreement that fibrillary aggregation of tau is the best correlator with the onset and progression of dementia. It is mostly accepted that abnormal posttranslational modifications, that is, hyperphosphorylation, acetylation, glycation, nitration, truncation, and others, are responsible for altered tau structure in AD. Some of these events have been sequentially staged during the formation of NFTs and the evolution of the disease. Validation at clinicopathological levels with the load of abnormally phosphorylated and truncated tau has been demonstrated in populations of AD cases. Particularly abnormal phosphorylation, acetylation, and truncation are further supported as pathological events by *in vitro* experiments demonstrating that these modifications increase fibrillization of tau and induce cell toxicity *in vitro*. Transgenic animals carrying these altered forms of tau protein also develop cognitive alterations. We believe that resolving the genesis of conformational changes of tau protein promoted by these posttranslational modifications and its role in fibrillization in disease are important achievements for assessing the potential of tau-directed therapies. Moreover, accurate determination of altered tau protein in the cerebrospinal fluid and other body fluids may provide better expectation to predict the onset and evolution of dementia.
